# Spatial relationships of intra-lesion heterogeneity in *Mycobacterium tuberculosis* microenvironment, replication status, and drug efficacy

**DOI:** 10.1371/journal.ppat.1010459

**Published:** 2022-03-28

**Authors:** Richard C. Lavin, Shumin Tan

**Affiliations:** 1 Department of Molecular Biology and Microbiology, Tufts University School of Medicine, Boston, Massachusetts, United States of America; 2 Graduate Program in Molecular Microbiology, Graduate School of Biomedical Sciences, Tufts University, Boston, Massachusetts, United States of America; National Institutes of Health, UNITED STATES

## Abstract

A hallmark of *Mycobacterium tuberculosis* (Mtb) infection is the marked heterogeneity that exists, spanning lesion type differences to microenvironment changes as infection progresses. A mechanistic understanding of how this heterogeneity affects Mtb growth and treatment efficacy necessitates single bacterium level studies in the context of intact host tissue architecture; however, such an evaluation has been technically challenging. Here, we exploit fluorescent reporter Mtb strains and the C3HeB/FeJ murine model in an integrated imaging approach to study microenvironment heterogeneity within a single lesion *in situ*, and analyze how these differences relate to non-uniformity in Mtb replication state, activity, and drug efficacy. We show that the pH and chloride environments differ spatially even within a single caseous necrotic lesion, with increased acidity and chloride levels in the lesion cuff versus core. Strikingly, a higher percentage of Mtb in the lesion core versus cuff were in an actively replicating state, and correspondingly active in transcription/translation. Finally, examination of three first-line anti-tubercular drugs showed that isoniazid efficacy was conspicuously poor against Mtb in the lesion cuff. Our study reveals spatial relationships of intra-lesion heterogeneity, sheds light on important considerations in anti-tubercular treatment strategies, and establishes a foundational framework for Mtb infection heterogeneity analysis at the single bacterium level *in situ*.

## Author summary

A critical aspect of tuberculosis disease, caused by the bacterium *Mycobacterium tuberculosis* (Mtb), is non-uniformity in bacterial physiology and disease progression. This heterogeneity is thought to be a major reason for the need for prolonged treatment, and understanding what and how facets of the infection differ within a host or single lesion is thus vital for the development of improved therapeutic strategies. However, elucidation and direct demonstration of bacterial non-uniformity during whole animal infection, in the context of intact tissue architecture, has been extremely challenging and consequently lacking. We show here using an integrated imaging approach that Mtb experiences different pH and chloride microenvironments depending on its sublocation within a structured lesion, which strikingly correlates with Mtb replication status and transcriptional/translational activity. We further find that these spatial differences impact critically on drug efficacy, with the first-line anti-tubercular drug isoniazid demonstrating conspicuously poor efficacy against Mtb residing in the structured lesion cuff, versus the bacteria present in the lesion core. Our study reveals how several aspects of non-uniformity in Mtb infection biology are related to spatial location within a structured lesion, and lays the groundwork for analysis of Mtb infection heterogeneity at the single bacterium level in intact tissue.

## Introduction

The ability to effectively treat *Mycobacterium tuberculosis* (Mtb) is significantly impeded by the marked heterogeneity of the infection across multiple levels, including non-uniformity in local microenvironments [1–7]. This heterogeneity extends not just between lesions but within a single lesion; for example, matrix-assisted laser desorption/ionization imaging mass spectrometry studies have demonstrated variation in drug penetration into caseous necrotic lesions [8,9], and the organization of immune cells and mediators are spatially distinct in these structured lesions [6,7]. Further, pH measurements of caseum dissected from caseous necrotic lesions in both C3HeB/FeJ mice and guinea pigs have indicated its neutral pH [8,10], which has been contrasted with the slightly acidic pH of the macrophage intraphagosomal environment that is a major niche of Mtb [11–13]. Of note, the first-line anti-tubercular drug pyrazinamide (PZA) shows increased efficacy in acidic conditions [14–16]. The neutral pH of the caseous material has thus been implicated as a contributing reason for the lack of PZA efficacy sometimes observed in C3HeB/FeJ mice where caseous necrotic lesions are formed, versus the uniform efficacy observed in BALB/c mice, which do not form such lesions [8,10].

The critical impact of within-host heterogeneity on infection and treatment outcome dictates the need to functionally characterize Mtb infection *in vivo* at the single bacterium level, within spatial tissue context. However, the technical hurdles associated with accomplishing such studies has meant a continued dearth in our knowledge of what Mtb actually “sees” during infection at the single bacterium level. Here, we establish an integrated imaging approach that overcomes technical challenges to address key questions regarding Mtb infection heterogeneity, including elucidation of how Mtb replication status differs spatially within a single lesion. We focus here on the caseous necrotic lesion core versus the cuff, given the distinct environments they represent for the bacteria, with Mtb residing predominantly extracellularly in the lesion core, versus within macrophages in the lesion cuff [17]. In particular, we show how local pH and chloride environments experienced by Mtb, and the replication status and transcriptional/translational activity of the bacterial population, differs in the caseous necrotic lesion core versus cuff. Examination of the impact of this spatial non-uniformity on the efficacy of anti-tubercular drugs further reveals a striking difference in efficacy of isoniazid against Mtb residing in the caseous necrotic lesion core versus cuff. Our study sheds light on spatial heterogeneity in Mtb physiology *in vivo* and lays the essential groundwork for single bacterium resolution *in situ* analysis of Mtb infection heterogeneity, knowledge vital for illuminating fundamental aspects of Mtb-host interactions and the development of new anti-tubercular treatment strategies.

## Results

### Visualizing Mtb infection at the single bacterium level within the context of intact tissue architecture

**Fig 1:**
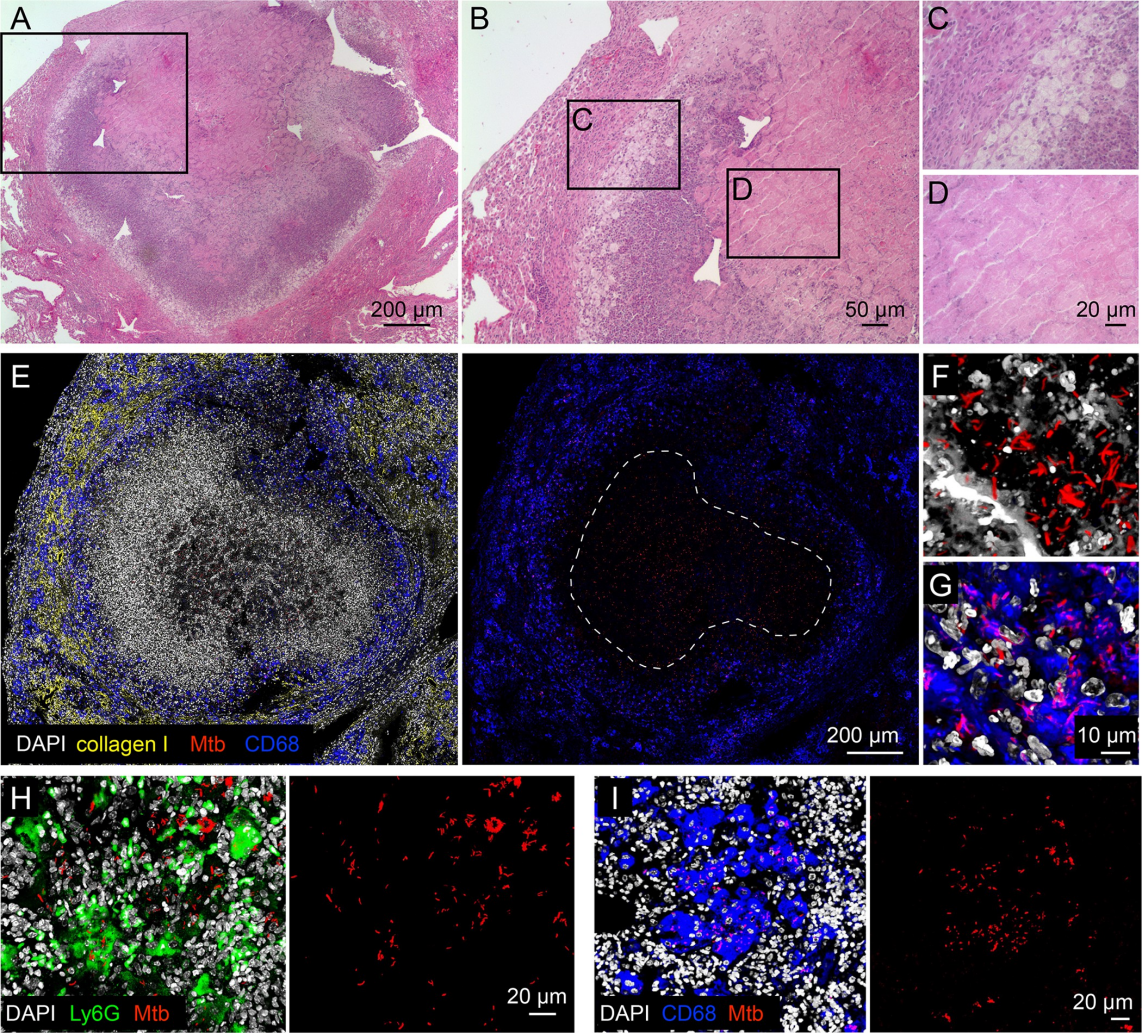
Visualizing Mtb infection at the single bacterium level within the context of intact tissue architecture. (A-D) Hematoxylin and eosin histology images of a type I caseous necrotic lesion from a six week infection of C3HeB/FeJ mice with Erdman (*smyc’*::mCherry). (B) shows the magnified view of the boxed region in (A), and (C) and (D) show magnified views of the respectively labeled boxed regions in (B). (E-G) Confocal images of a type I caseous necrotic lesion (~6 x 6 tiled image) from a six week infection of C3HeB/FeJ mice with Erdman (*smyc’*::mCherry). All bacteria are marked in red (*smyc’*::mCherry), nuclei are shown in grayscale (DAPI), collagen I is shown in yellow, and macrophages are shown in blue (CD68). The right panel in (E) shows just the CD68 and *smyc’*::mCherry signal, with the lesion core outlined. (F) and (G) are high magnification 3D confocal images from the lesion core and cuff respectively. (H) 3D confocal images of a type II neutrophil-dominant lesion (~3 x 3 tiled image) from a six week infection of C3HeB/FeJ mice with Erdman (*smyc’*::mCherry). All bacteria are marked in red (*smyc’*::mCherry), nuclei are shown in grayscale (DAPI), and neutrophils are shown in green (Ly6G). The panel on the right shows just the *smyc’*::mCherry signal. (I) Confocal images of a type III macrophage-dominant lesion (~3 x 3 tiled image) from a six week infection of C3HeB/FeJ mice with Erdman (*smyc’*::mCherry). All bacteria are marked in red (*smyc’*::mCherry), nuclei are shown in grayscale (DAPI), and macrophages are shown in blue (CD68). The panel on the right shows just the *smyc’*::mCherry signal.

Interrogating the spatial relationships between the local environment and Mtb replication status first requires an integrated imaging approach that enables analysis of individual lesions within an infected lung. To establish this approach, we infected C3HeB/FeJ mice with Mtb constitutively expressing mCherry and harvested the lungs six weeks post-infection (S1 Fig). The utility of C3HeB/FeJ mice as a Mtb infection model has been increasingly appreciated due to its formation of a range of lesion types including caseous necrotic lesions, and it is now frequently used in anti-tubercular drug studies [8,10,18–23]. By employing a broad xy-plane tiled imaging approach coupled with antibody staining against host markers, we were able to distinguish the three lesion types previously described via histological studies in the C3HeB/FeJ murine Mtb infection model [4,17]. Of particular interest here, highly structured type I caseous necrotic lesions that contain a necrotic core ringed by foamy macrophages, as observed by histological hematoxylin and eosin staining (Fig 1A–1D) [17], were distinguished in confocal microscopy imaging by the fibrous collagen I-rich cuff containing CD68-positive macrophages that rings the caseous necrotic core (Fig 1E–1G). Very rare neutrophil-dominant type II lesions were discriminated by Ly6G staining for neutrophils (Fig 1H), while macrophage-rich type III lesions were differentiated with CD68 staining of macrophages (Fig 1I). Broad xy-plane tiled imaging provides the breadth required to capture large type I lesions in their entirety (Fig 1E), with subsequent targeted 3-dimensional imaging and reconstruction enabling single cell-resolution visualization of the fluorescent bacteria (Fig 1F and 1G), setting the stage for analysis of intra-lesion sublocation environment and Mtb replication status.

We focus here on type I caseous necrotic lesions, due to its association with heterogeneity in drug penetration and Mtb drug response [8–10,18–20,24], and its highly structured nature.

### pH and Cl^-^ microenvironment experienced by Mtb differs in the cuff versus core of caseous necrotic lesions

**Fig 2:**
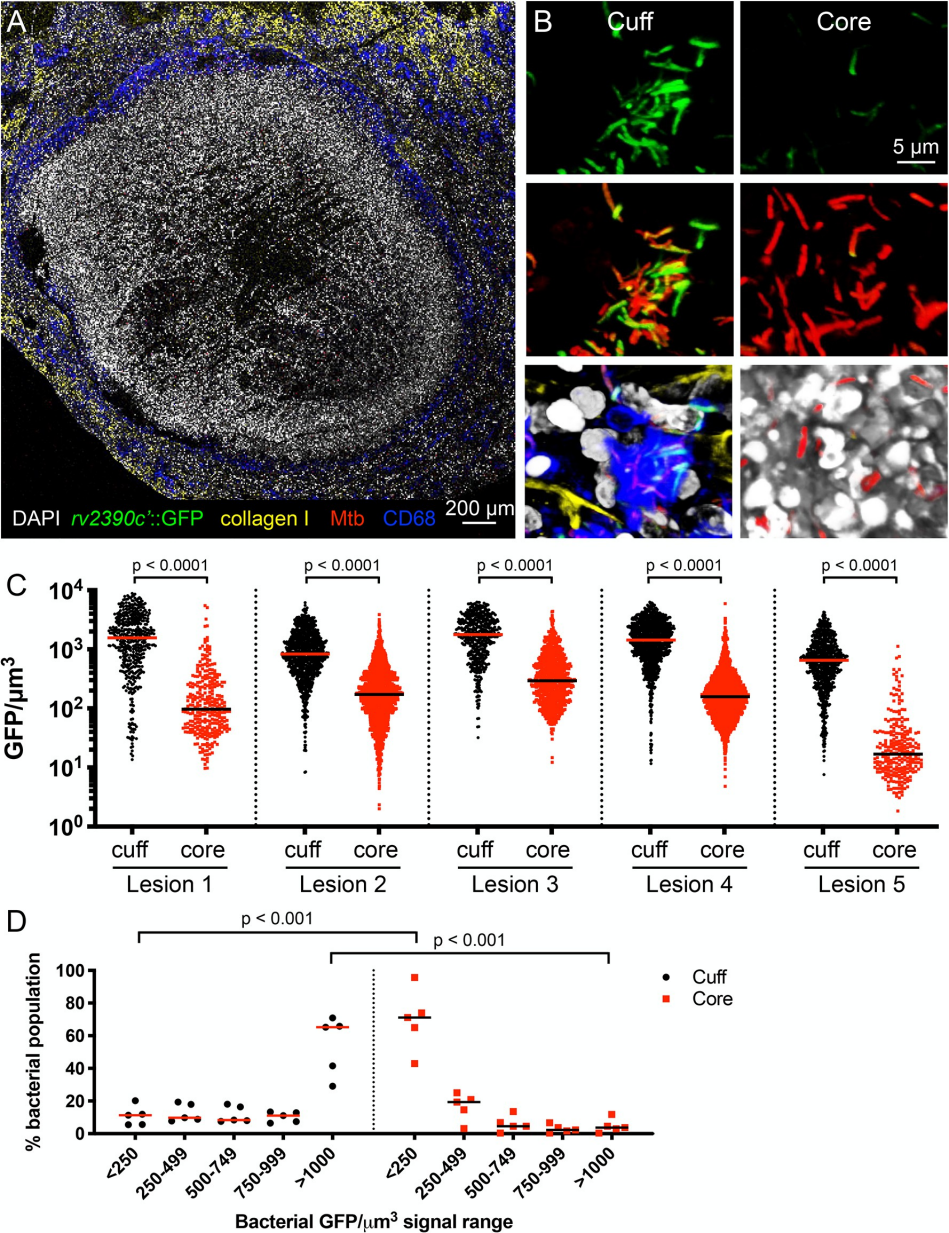
pH and Cl^-^ microenvironment experienced by Mtb differs in the cuff versus core of caseous necrotic lesions. (A and B) Confocal microscopy images of type I caseous necrotic lesions from 6–8 week infection of C3HeB/FeJ mice with Erdman (*rv2390c’*::GFP, *smyc’*::mCherry). Overview image of a lesion (~10 x 11 tiled image) is shown in (A), and representative 3D confocal images from the lesion cuff and core shown in (B). All bacteria are marked in red (*smyc’*::mCherry), reporter signal is shown in green (*rv2390c’*::GFP), nuclei are shown in grayscale (DAPI), collagen I is shown in yellow, and macrophages are shown in blue (CD68). (C) shows *rv2390c’*::GFP/μm^3^ signal for individual bacteria or a group of tightly clustered bacteria, quantified from multiple 3D confocal images at each lesion sublocation (5 different lesions from 5 mice; number of bacteria quantified was respectively 478, 307, 937, 1715, 516, 885, 1078, 1755, 749, and 257 for each lesion sublocation as shown from left to right on the graph). Horizontal lines mark the median value for each sample. p-values were obtained with a Mann-Whitney statistical test. (D) shows data from (C) binned into 5 sub-ranges of GFP/μm^3^ signal. Each point on the graph represents one lesion. Horizontal lines mark the median value for each group. p-values were obtained with a multiple t-test with a Holm-Sidak correction.

With the infection and imaging approach established, we next sought to directly visualize differences in the pH and chloride (Cl^-^) microenvironment within type I caseous necrotic lesion sublocations, given the reported neutral pH of dissected caseum [8,10], and our previous work demonstrating the synergistic transcriptional response of Mtb upon exposure to acidic pH and high Cl^-^ levels, which are linked cues during macrophage phagosomal maturation [13]. To do so, we infected C3HeB/FeJ mice with our pH/Cl^-^-responsive fluorescent reporter Mtb strain (Erdman *rv2390c’*::GFP, *smyc’*::mCherry) that fluoresces green upon bacterial exposure to acidic pH and/or high Cl^-^ levels in the local environment, and expresses mCherry constitutively for visualization of all Mtb irrespective of local environment [13,25]. Comparison of Mtb present in the lesion cuff (predominantly present within macrophages) versus those present in the necrotic lesion core showed that the *rv2390c’*::GFP reporter signal was significantly higher in the bacteria present in the lesion cuff (Fig 2A–2C), even as non-uniformity in reporter signal within each sublocation remained notable (Fig 2C). Still more strikingly, binning the data for each lesion into different *rv2390c’*::GFP reporter signal ranges demonstrated the opposite distributions in physiological cues experienced by the two bacterial populations (Fig 2D). Specifically, most Mtb present in the lesion cuff expressed high levels of *rv2390c’*::GFP reporter fluorescence (Fig 2B–2D), indicative of an environment with more acidic pH and/or higher [Cl^-^], and in accord with the environment that would be expected in macrophage phagosomes [13]. Conversely, a majority of bacteria present in the necrotic core (extracellular Mtb) expressed lower levels of *rv2390c’*::GFP reporter signal (Fig 2B–2D), indicative of an environment that is at a more neutral pH/has a lower [Cl^-^].

Our findings demonstrate at the single bacterium level within intact tissue that (i) the pH and chloride environment of the caseous necrotic core significantly differs from that experienced by Mtb present within the lesion cuff, and (ii) there remains non-uniformity in the local environment within each sublocation, even in the necrotic core.

### Mtb replication status differs in the cuff versus core of caseous necrotic lesions

**Fig 3:**
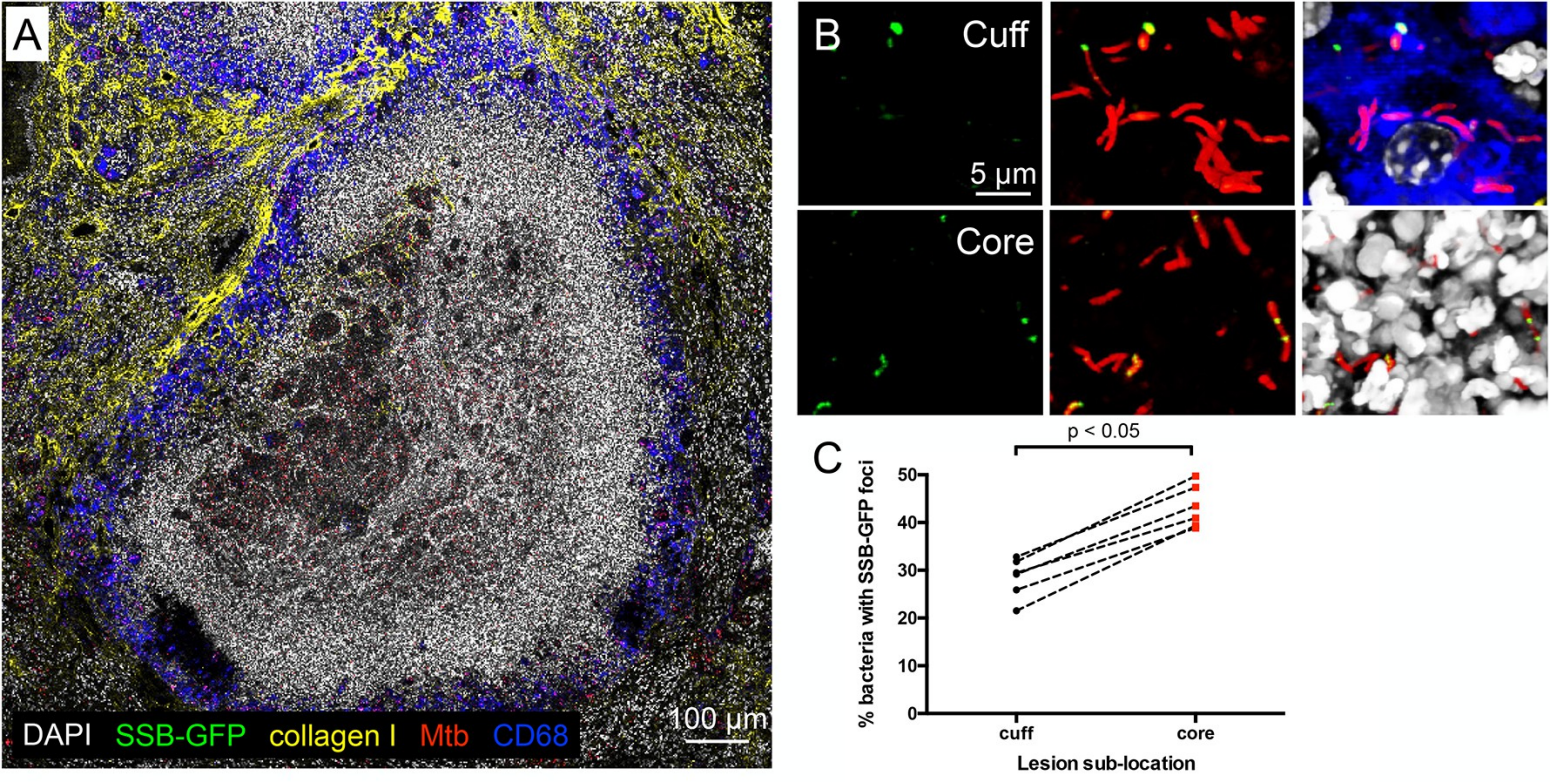
Mtb replication status differs in the cuff versus core of caseous necrotic lesions. (A and B) Confocal microscopy images of type I caseous necrotic lesions from 6 week infection of C3HeB/FeJ mice with Erdman (SSB-GFP, *smyc’*::mCherry). Overview image of a lesion (~5 x 5 tiled image) is shown in (A), and representative 3D confocal images from the lesion cuff and core are shown in (B). All bacteria are marked in red (*smyc’*::mCherry), reporter signal is shown in green (SSB-GFP), nuclei are shown in grayscale (DAPI), collagen I is shown in yellow, and macrophages are shown in blue (CD68). Panels with different combination of colors shown are presented from left to right for the cuff and core region in (B). For clarity of foci visualization, SSB-GFP signal is shown in extended focus, overlaid on the 3D images in (B). (C) shows the percentage of Mtb displaying SSB-GFP foci in each lesion sublocation for each quantified lesion measured from multiple 3D confocal images. Dashed line connects the data for the cuff and core of one lesion. (6 different lesions from 5 mice; number of bacteria quantified in each lesion sublocation [cuff, core] was [678, 475], [564, 545], [370, 446], [478, 1248], [537, 867], and [393, 360]). p-value was obtained using a Wilcoxon matched-pairs signed rank test.

To understand how this intra-lesion heterogeneity in microenvironment affects Mtb infection outcome, we next utilized our previously described single-strand DNA-binding protein (SSB)-GFP replication reporter to determine the replication status of Mtb in sublocations within a lesion. In this reporter strain, the Mtb SSB protein is translationally fused to GFP, driven by the native *ssb* promoter, and Mtb undergoing active DNA replication exhibit green foci, providing a proxy for revealing the replication status of a given bacterium [25,26]. Strikingly, analysis of lung tissue from C3HeB/FeJ mice infected with this reporter Mtb strain showed that a significantly greater percentage of Mtb present in the lesion core possessed SSB-GFP foci, indicative of actively replicating bacteria, versus Mtb present in the lesion cuff (Fig 3, compare cuff versus core in Fig 3B and 3C). This difference in overall replication status between Mtb present in the two lesion sublocations fits with the observed differences in local pH/[Cl^-^] (Fig 2), with a higher percentage of replicating Mtb in the less harsh environment of the necrotic core.

The replication state of Mtb can differentially impact drug efficacy and is implicated as a major contributor to the difficulties of successfully treating Mtb infection, as well as to the necessity for a prolonged treatment time course [27–31]. Yet actual demonstration of how Mtb replication status may differ within a single host during infection has been difficult to establish. Our data presented here provides the first direct evidence, to our knowledge, of how Mtb replication status differs not just within a single host but within a single lesion, and reveals how the non-uniformity in intra-lesion Mtb growth status is spatially related to lesion architecture.

### Transcriptional/translational activity of Mtb differs depending on bacterial location within caseous necrotic lesions

**Fig 4:**
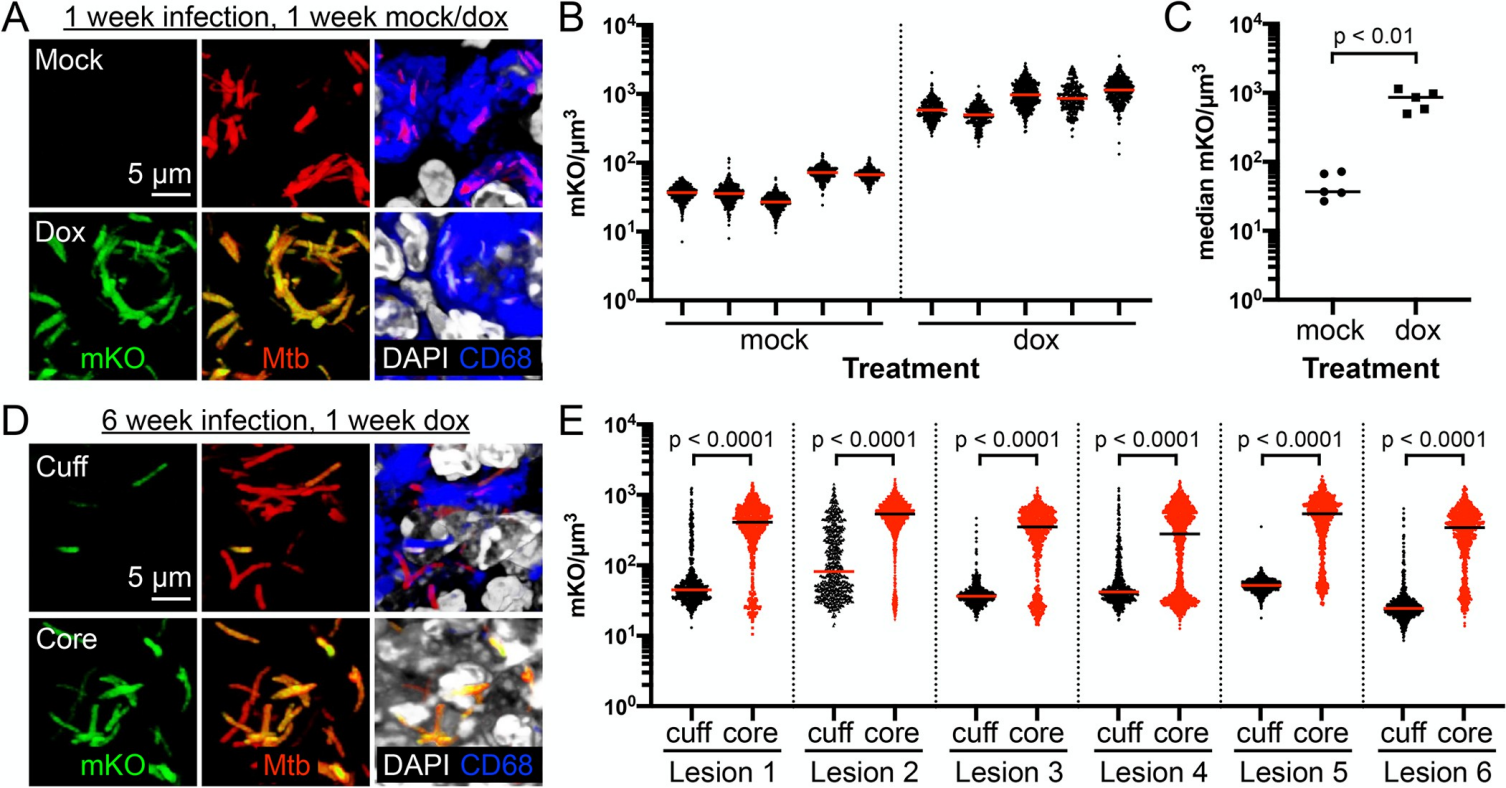
Heterogeneity in bacterial transcriptional/translational activity within different sublocations of caseous necrotic lesions. (A) Representative 3D confocal images from a 1 week infection of C3HeB/FeJ mice with Erdman (P_606_’::mKO-tetON, *smyc’*::mCherry), followed by 1 week of exposure to drinking water ± 1 mg/ml doxycycline. All bacteria are marked in red (*smyc’*::mCherry), reporter signal is shown in green (P_606_’::mKO-tetON), nuclei are shown in grayscale (DAPI), and macrophages are shown in blue (CD68). (B) mKO/μm^3^ signal for individual bacteria or a group of tightly clustered bacteria, quantified from multiple 3D confocal images from infections as performed in (A) (5 different mice/treatment group; number of bacteria quantified from each sample was respectively 440, 424, 604, 379, 436, 355, 330, 545, 257, and 425 as shown from left to right on the graph). Horizontal lines mark the median value for each sample. (C) graphs the medians of each sample shown in (B), with p-value obtained with a Mann-Whitney statistical test. (D) Representative 3D confocal images from the lesion cuff and core from a 6 week infection of C3HeB/FeJ mice with Erdman (P_606_’::mKO-tetON, *smyc’*::mCherry), followed by 1 week of exposure to drinking water + 1 mg/ml doxycycline. All bacteria are marked in red (*smyc’*::mCherry), reporter signal is shown in green (P_606_’::mKO-tetON), nuclei are shown in grayscale (DAPI), and macrophages are shown in blue (CD68). (E) mKO/μm^3^ signal for individual bacteria or a group of tightly clustered bacteria, quantified from multiple 3D confocal images at each lesion sublocation (6 different lesions from 5 mice; number of bacteria quantified was respectively 612, 745, 728, 1802, 488, 913, 636, 1615, 1527, 1058, 599, and 921, for each lesion sublocation as shown from left to right on the graph). Horizontal lines mark the median value for each sample. p-values were obtained with a Mann-Whitney statistical test.

The marked difference in bacterial replication state of Mtb residing in the caseous necrotic lesion core versus cuff prompted us to test a second, independent, approach to analyzing Mtb physiological state *in situ*. A dual fluorescent system where one fluorophore is placed under the control of an inducible promoter and a second spectrally distinct fluorophore is expressed constitutively has been utilized in differentiating transcriptionally active versus non-active Mtb in cultured macrophages [32–34]. We thus applied this strategy here *in vivo*, exploiting a reporter Mtb strain that carries on the chromosome a tetracycline inducible monomeric Kusabira Orange (mKO) construct, along with a constitutively expressed mCherry (P_606_’::mKO-tetON, *smyc’*::mCherry) [35]. As an initial test of the system, we infected C3HeB/FeJ mice with the Erdman (P_606_’::mKO-tetON, *smyc’*::mCherry) reporter Mtb strain for one week, before the provision of drinking water containing 5% sucrose ± 1 mg/ml doxycycline (dox) for one additional week. As shown in Fig 4A–4C, dox treatment in this short-term infection resulted in the expected induction of mKO fluorescence in Mtb, with Mtb in the mock-treated mice displaying no mKO fluorescence, in accord with results observed with this reporter in C57BL/6J mice [35], and reinforcing the suitability of dox induction for *in vivo* Mtb studies [35,36].

**Fig 5:**
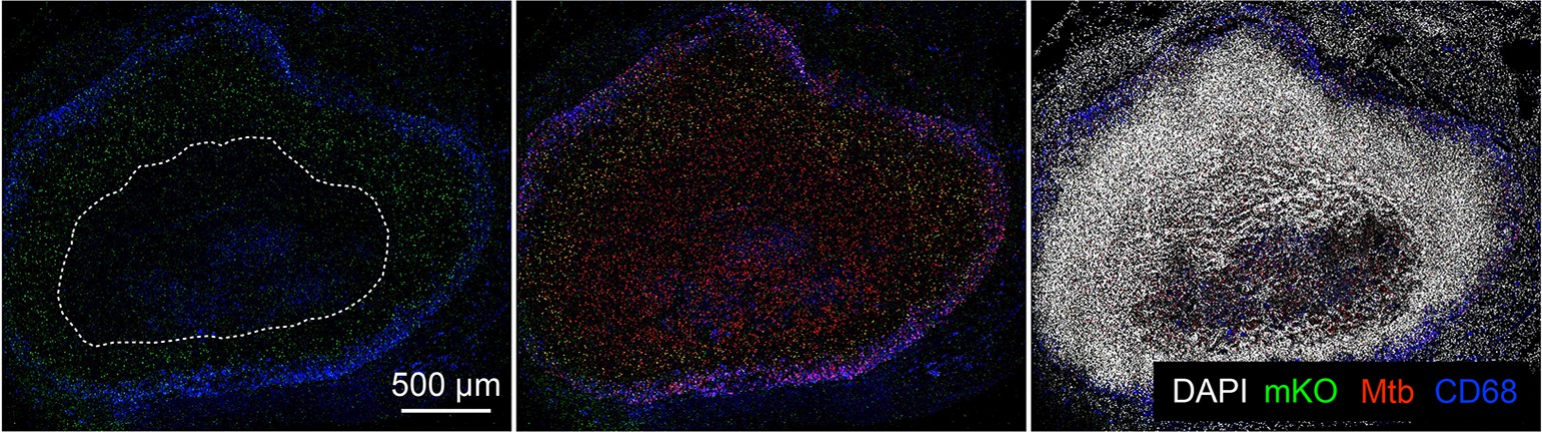
Inhibition of doxycycline penetration into the very central region of caseous necrotic lesions. Overview confocal image (~11 x 9 tiled image) from a 6 week infection of C3HeB/FeJ mice with Erdman (P_606_’::mKO-tetON, *smyc’*::mCherry), followed by 1 week of exposure to drinking water + 1 mg/ml doxycycline. All bacteria are marked in red (*smyc’*::mCherry), reporter signal is shown in green (P_606_’::mKO-tetON), nuclei are shown in grayscale (DAPI), and macrophages are shown in blue (CD68). The dashed line demarcates the central core region where no mKO signal is observed.

To assess spatial intra-lesion differences in Mtb transcriptional/translational activity, we infected C3HeB/FeJ mice with this Erdman (P_606_’::mKO-tetON, *smyc’*::mCherry) reporter Mtb strain, allowing the infection to establish for six weeks prior to a one week exposure of the mice to drinking water containing 5% sucrose ± 1 mg/ml dox, and harvesting of lung tissue. A first observation revealed by broad xy-plane imaging was that penetration of dox into the very central core of the caseous necrotic lesion appeared impeded, as no mKO signal could be observed in Mtb present there (Fig 5). Nonetheless, as mKO signal could be observed within the more peripheral regions of the caseous necrotic core (Fig 5), analysis of the differences in mKO induction in Mtb present in the caseous necrotic core versus cuff was still feasible. Conspicuously, induction of mKO signal was seen in a much greater percentage of Mtb present in the peripheral regions of the caseous necrotic core versus those in the lesion cuff (Fig 4D and 4E). Additionally, mKO signal induction variation within both the cuff and core Mtb populations was significantly greater than that observed in the short-term infection (compare the range within each scatter plot in Fig 4B and 4E). These data are consistent with the differences in bacterial replication status between Mtb present in the caseous necrotic core versus cuff, and further supports and highlights the impact of lesion sublocation on Mtb physiological state.

### Some first-line anti-tubercular drugs exhibit altered efficacy against Mtb present in the cuff versus core of caseous necrotic lesions

**Fig 6:**
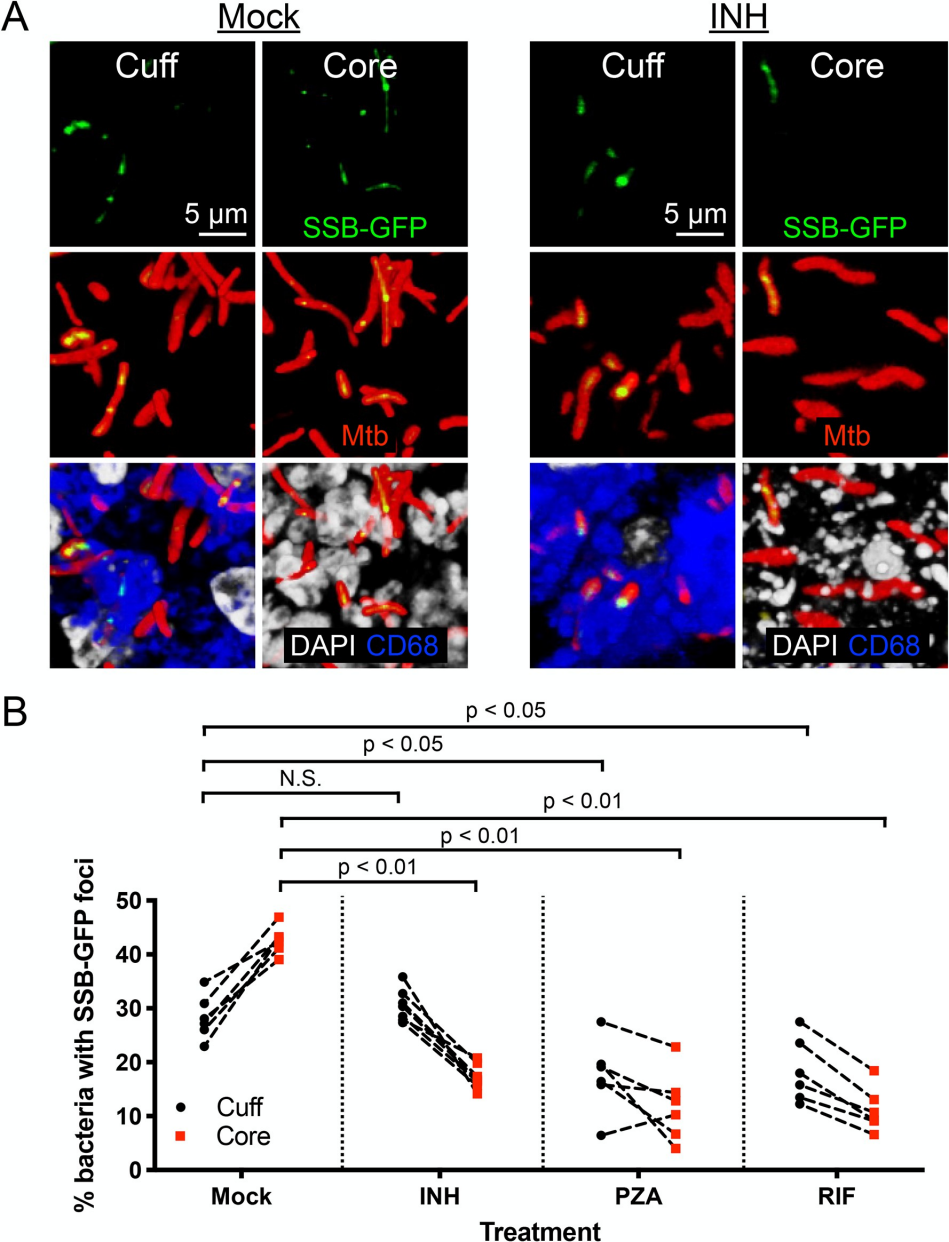
Impact of bacterial sublocation within caseous necrotic lesions on efficacy of first-line anti-tubercular drugs. (A) Representative 3D confocal images from the lesion cuff and core from a 6 week infection of C3HeB/FeJ mice with Erdman (SSB-GFP, *smyc’*::mCherry), followed by 2 weeks of mock or 10 mg/kg isoniazid (INH) treatment. All bacteria are marked in red (*smyc’*::mCherry), reporter signal is shown in green (SSB-GFP), nuclei are shown in grayscale (DAPI), and macrophages are shown in blue (CD68). For clarity of foci visualization, SSB-GFP signal is shown in extended focus, overlaid on the 3D images. (B) shows the percentage of Mtb displaying SSB-GFP foci in each lesion sublocation for each quantified lesion, measured from multiple 3D confocal images for each set of 6 week infections followed by 2 weeks of mock, 10 mg/kg INH, 150 mg/kg pyrazinamide (PZA), or 10 mg/kg rifampicin (RIF) treatment. p-values were obtained with a Mann-Whitney statistical test. Dashed line connects the data for the cuff and core of one lesion. Sample details are as follows: mock treatment set– 6 different lesions from 5 mice; number of bacteria quantified in each lesion sublocation [cuff, core] was [641, 891], [481, 389], [408, 339], [318, 367], [447, 425], and [541, 453]. INH treatment set– 7 different lesions from 4 mice; number of bacteria quantified in each lesion sublocation [cuff, core] was [319, 371], [371, 467], [417, 385], [450, 403], [412, 419], [339, 311], and [368, 339]. PZA treatment set– 6 different lesions from 5 mice; number of bacteria quantified in each lesion sublocation [cuff, core] was [219, 210], [423, 626], [418, 543], [248, 419], [285, 366], and [341, 375]. RIF treatment set– 6 different lesions from 4 mice; number of bacteria quantified in each lesion sublocation [cuff, core] was [215, 297], [300, 151], [246, 275], [212, 122], [318, 378], and [367, 215].

The observed differences in pH/Cl^-^ environment and in replication status and transcriptional/translational activity between Mtb residing in the lesion core versus cuff raised the question of whether anti-tubercular drugs may have differential efficacy even within a single caseous necrotic lesion, separate from lesion penetration issues. To address this question, we infected C3HeB/FeJ mice for six weeks with the Erdman (SSB-GFP, *smyc’*::mCherry) reporter Mtb strain, before starting treatment via oral gavage with isoniazid (INH), pyrazinamide (PZA), or rifampicin (RIF) for two weeks. These three first-line drugs were chosen for their known ability to penetrate well into the core of caseous necrotic lesions, as demonstrated by matrix-assisted laser desorption/ionization imaging mass spectrometry (MALDI-IMS), unlike other anti-tubercular drugs such as bedaquiline and moxifloxacin [8,9,37]. Prominently, consistent with the better efficacy of INH against actively replicating bacteria [38–40], INH treatment was significantly more effective against Mtb present in the caseous necrotic core versus the cuff, with a decrease in the percentage of Mtb with SSB-GFP foci compared to the mock-treated mice in the lesion core (Fig 6, compare mock versus INH-treated core images in Fig 6A, and quantification in Fig 6B). In contrast, no significant difference in the percentage of Mtb with SSB-GFP foci was observed between the INH and mock-treated groups in the lesion cuff (Fig 6, compare mock versus INH-treated cuff images in Fig 6A, and quantification in Fig 6B).

In the case of PZA, treatment decreased the percentage of actively replicating Mtb in both lesion sublocations compared to the mock-treated control (Fig 6B). While PZA has been shown to be more efficacious at acidic versus neutral pH [15,16], and the caseous necrotic core has a more neutral pH (Fig 2) [8,10], it is now also appreciated that PZA can have efficacy in conditions that occur at neutral pH [41,42]. Our observations here support the concept that PZA has efficacy even within the caseous necrotic core, suggesting that the *in vivo* effect of PZA extends beyond a dependency on local acidic pH conditions.

Finally, while RIF treatment showed efficacy against the Mtb population present in both lesion sublocations, it decreased the percentage of Mtb with SSB-GFP foci in the caseous necrotic core to a greater extent (Fig 6B). RIF acts to inhibit Mtb transcription [43–45], and our findings here are thus in accord with its mode of action. Overall, these results demonstrate how efficacy of different anti-tubercular drugs can vary within a single caseous necrotic lesion, separate from questions of lesion penetration, and in correlation with bacterial replication and physiological state.

## Discussion

Despite the consensus on the importance of heterogeneity on Mtb infection progression and treatment outcome, population-level readouts such as bacterial load and host cytokine levels continue to be the primary means by which these outcomes are measured. Our establishment here of an integrated imaging approach for *in situ* tissue analysis presents a method to overcome the significant hurdle of the single bacterium resolution required for analysis of Mtb *in vivo* infection heterogeneity from the bacterial perspective. We delineate pH and Cl^-^ as two facets of the microenvironment that exhibit intra-lesion heterogeneity during Mtb infection, findings that expand on previous studies reporting on the neutral pH of dissected caseum [8,10], by providing single bacterium resolution analysis in both the lesion cuff and core sublocations, and revealing that heterogeneity in pH and [Cl^-^] is additionally present even within a lesion sublocation. The local pH and [Cl^-^] environment experienced by intracellular Mtb within the lesion cuff is expected to be driven by the actions of both the host and the bacterium, given the decrease in pH and increase in [Cl^-^] that occur during macrophage phagosome maturation [13], and the active inhibition of aspects of phagosome maturation by Mtb, such as via inhibition of the vacuolar-H^+^-ATPase [46]. The variation in pH and [Cl^-^] in the local environment still observed even within the population of Mtb residing in the necrotic core was not correlated with specific sub-regions of the core (e.g. there was no distinction in reporter signal for Mtb in the very center of the lesion core versus the more peripheral regions of the core), and whether these differences have active drivers will require further investigation.

We anticipate that other vital environmental signals such as nitric oxide, which is actively produced by host immune cells, are likely to also exhibit intra-lesion heterogeneity. In addition, pimonidazole-based histological labeling has indicated the presence of hypoxia in the cuff of caseous necrotic lesions [24,47]. Reduction of pimonidazole for labeling requires the activity of host nitroreductases, and it has thus been assumed that hypoxia also exists in the very central core of the lesion, with the lack of viable host cells in the necrotic lesion core accounting for the lack of pimonidazole labeling [48]. Future studies utilizing reporters of Mtb hypoxia and NO response [13,25], and to other environmental cues such as iron and potassium [49,50], hold potential for verifying these assumptions, and in building on the framework understanding established here of the relationship between local environment in the lesion core versus cuff and Mtb replication state.

Excitingly, utilization of the SSB-GFP replication reporter enabled, to our knowledge, the first *in vivo* and *in situ* comparison of the replication state of Mtb present in the caseous necrotic core versus in macrophages in the lesion cuff, with a higher percentage of Mtb in the lesion core versus cuff found to be in an actively replicating state. While this fits with the less acidic pH/lower [Cl^-^] microenvironment of the lesion core versus cuff, the result is intriguing in the context of *ex vivo* experiments with caseum Mtb from a rabbit infection model that reported decreased replication state of the bacteria [51]. A key distinction to note here is that the comparison made in our studies is of Mtb replication status in the caseous necrotic lesion core versus the cuff in their *in situ* and *in vivo* context, and not against bacteria in broth or *in vitro* macrophage infection conditions. Indeed, the percentage of Mtb residing in the necrotic lesion core undergoing active DNA replication is lower than what is observed for bacteria grown in broth (76–80% for Mtb in broth) [25]. Our studies here have also focused on one time point (6 weeks), which represents an early, first establishment stage of caseous necrotic lesions in the C3HeB/FeJ murine model utilized [17]. In contrast, the *ex vivo* caseum study utilized caseum extracted from lesions in rabbits that had been infected for 12 to 16 weeks [51]. It will thus be crucial in future studies to develop reporters that can be stably maintained for longer-term *in vivo* infections, to delineate if the conditions within the caseous necrotic core change with time and infection state. Additional analyses expanding on the reporters utilized to encompass those that report on Mtb metabolism, such as cholesterol utilization [52], will further provide critical insight into differences in the environment and physiological state of Mtb in the necrotic core versus the lesion cuff. Parallel time course studies combining the replication and/or transcriptional/translational reporter with drug treatments will similarly be important in delineating if drug efficacy against Mtb residing in the necrotic core versus lesion cuff change with time and infection state.

Complementary to the SSB-GFP replication reporter, the P_606_’::mKO-tetON, *smyc’*::mCherry reporter provided a method for analyzing bacterial transcriptional/translational activity *in vivo* and *in situ*. While we posit that the lack of mKO signal seen from bacteria in the very central core of the caseous necrotic lesion arises from impedance of dox penetration into the lesion core, it will be interesting in future studies to directly examine dox penetration into caseous necrotic lesions with fine spatial resolution, using methods such as MALDI-IMS or fluorescent dox derivatives. A previous study utilizing laser capture microdissection from Mtb-infected rabbits had reported that dox concentrations in the caseum were comparable to those from the cellular region surrounding the necrotic core, but did not examine for possible differences in dox levels between the peripheral regions of the necrotic core versus the very central region [53,54]. These studies also showed significant variation between lesions, with caseum/cellular dox concentration <1 in 9/16 lesions examined (low of 0.3), and further used a significantly higher dose of dox than utilized in the present study (2000 ppm supplemented in food, versus 1 mg/ml in water here, which is ~1333 ppm in food, extrapolating from data in Redelsperger et al [55]). It is possible that utilizing a higher dose of dox will enable increased penetration, and future studies testing for correlation between dox dose and the depth into the lesion core at which induction of mKO signal from P_606_’::mKO-tetON, *smyc’*::mCherry reporter Mtb can be observed will be an additional method for probing dox lesion penetration characteristics.

By elucidating and directly demonstrating the spatial non-uniformity in Mtb replication status and transcriptional/translational activity within a single lesion and its striking correlation to local differences in drug efficacy, this work sets a foundational framework for interrogation of: (i) the heterogeneity in diverse aspects of Mtb infection biology within and between lesions in a single host, via the use of various reporter Mtb strains [49,50,52,56–58], (ii) how drug treatment efficacy may differ in different sublocations within the lung/lesions and/or affect the local environment experienced by the bacterium, and (iii) how targeting of Mtb response to the local environment may change the extent of heterogeneity observed and thereby alter treatment success. We propose that future such single bacterium level studies in the context of intact tissue architecture, perturbing either regulators of bacterial environmental sensing and response or testing the effect of various therapeutic combinations, will build on the groundwork laid here and provide critical insight into what drives infection heterogeneity, and how such non-uniformity impacts our ability to successfully treat Mtb infection.

Finally, the approaches and concepts established here are likely to also have broad applicability to other bacterial species, with the burgeoning appreciation of the importance of spatial heterogeneity in varied aspects of bacterial-host interactions for infection outcome of many pathogens. This spans spatial differences in *Staphylococcus aureus* biology in abscesses and *Yersinia pseudotuberculosis* physiology and host response within microcolonies [59–62], to microniches in the gastrointestinal tract and its implications for bacterial survival and growth [63,64]. Spatial differences within biofilms of bacteria such as *Pseudomonas aeruginosa* and *Vibrio cholerae* have also recently been elegantly demonstrated [65,66]. Extension of our approaches to examine bacterial replication and activity state at the single cell level *in situ* to other bacterial systems thus hold exciting potential for further revealing the impact of spatial heterogeneity during infection on pathogen growth and treatment success.

## Materials and methods

### Ethics statement

All animal procedures followed standards set by the National Institutes of Health “Guide for the Care and Use of Laboratory Animals”. Animal protocols were reviewed and approved by the Institutional Animal Care and Use Committee at Tufts University (#B2021-139), in accordance with the Association for Assessment and Accreditation of Laboratory Animal Care, the US Department of Agriculture, and the US Public Health Service guidelines. Light anesthesia during infection and oral gavage administration of drugs was via exposure to 2% isoflurane delivered by a vaporizer system. Euthanasia utilized carbon dioxide gas with regulated flow, consistent with American Veterinary Medical Association guidelines.

### Mtb strains and culture

Reporter Mtb strains (*smyc’*::mCherry; *rv2390c’*::GFP, *smyc’*::mCherry; SSB-GFP, *smyc’*::mCherry) used for measuring differences in local microenvironment and replication status were in the Erdman background and have been previously described [13,25]. The P_606_’::mKO-tetON, *smyc’*::mCherry reporter has also been previously described [35]. Bacteria were cultured in standing T25 flasks with filter caps, in 7H9 Middlebrook medium supplemented with OADC, 0.05% Tween 80, and 50 μg/ml hygromycin B or 25 μg/ml kanamycin as needed, buffered at pH 7.0 with 100 mM MOPS. Preparation of Mtb stocks for mice infection were as previously described [25].

### Mouse Mtb infections

6–8 week old female C3HeB/FeJ wild type mice (Jackson Laboratory, Bar Harbor, ME) were intranasally infected with 10^3^ colony forming units of appropriate reporter Mtb strain in 35 μl of phosphate-buffered saline (PBS) containing 0.05% Tween 80, under light anesthesia with 2% isoflurane. Mice were sacrificed at 2, 6, 7, or 8 weeks post-infection, and the lungs fixed overnight in 4% paraformaldehyde (PFA) in PBS, before transfer and storage in PBS prior to analysis. For histology imaging, lung lobes were paraffin-embedded and processed for standard hematoxylin and eosin staining (Tufts Comparative Pathology Services), with images captured using a Nikon Eclipse E400 microscope equipped with a SPOT Insight color digital camera.

For the P_606_’::mKO-tetON, *smyc’*::mCherry reporter strain infection, infections were allowed to establish for one or six weeks, before provision of the mice with water containing 5% sucrose ± 1 mg/ml doxycycline, with one additional water change during the one week treatment period. For the drug treatment infections, C3HeB/FeJ wild type mice were infected with the SSB-GFP, *smyc’*::mCherry reporter and infection allowed to establish for six weeks, before commencement of treatment with 10 mg/kg isoniazid (INH), 150 mg/kg pyrazinamide (PZA), or 10 mg/kg rifampicin (RIF) via oral gavage five times a week for 2 weeks (in a 200 μl volume). Control infected mice were mock-treated with sterile water. All drugs were prepared weekly, and all oral gavage treatments were carried out under light anesthesia with 2% isoflurane. INH and PZA working solutions were prepared directly in sterile water. RIF working solution was prepared by diluting 50 mg/ml RIF stock in DMSO to 10 mg/kg RIF + 5% DMSO in sterile water.

### Confocal immunofluorescence microscopy

Fixed lung lobes were embedded in 4% agarose in PBS and 250 μm sections obtained with a Leica VT1000S vibratome [67]. Staining of tissue was essentially as previously described [13,25,67]–lung sections were blocked and permeabilized in PBS + 3% BSA + 0.1% Triton X-100 (“blocking buffer”) for 1 hour at room temperature, before incubation with primary antibodies overnight at room temperature (all steps on a nutator). The next morning, samples were washed 3 x 5 minutes with blocking buffer, then incubated with secondary antibodies at room temperature for 2 hours (all steps on a nutator). Samples were washed 3 x 5 minutes with blocking buffer again, and mounted with Vectashield mounting medium (Vector labs, Burlingame, CA). Rabbit anti-collagen I (Novus Biologicals, Centennial, CO, catalog #NB600-408) was used at 1:250, and Alexa Fluor 514 goat anti-rabbit (Invitrogen, Carlsbad, CA, catalog #A31558) used at 1:200 for secondary detection. Rat anti-CD68 (Bio-Rad, Hercules, CA, catalog #MCA1957) and rat anti-Ly6G (BD Biosciences, San Jose, CA, catalog #551459) were each used at 1:100, and Alexa Fluor 647 goat anti-rat (Invitrogen, catalog #A21247) used at 1:100 for secondary detection. Nuclei were visualized with DAPI (1:500; Invitrogen, catalog #D3571). Samples were imaged on a Leica SP8 spectral confocal microscope.

For broad xy-plane imaging, the Leica LAS X Navigator module was used to obtain multiple overlapping images that were then automatically merged together. High resolution images for reporter quantification were 10 μm in depth, reconstructed into 3D using Volocity software (Quorum Technologies, Ontario, Canada) from images taken at 0.5 μm z-steps. For all experiments except for those with the P_606_’::mKO-tetON, *smyc’*::mCherry reporter strain, high magnification and resolution images for reporter signal quantification for Mtb residing in the lesion core were taken both from the central core and the more peripheral regions of the core. Regions were selected based only on bacteria presence, and without regard for reporter signal. In each case, 3–6 images were taken, each with a 40x oil immersion objective at 2x digital zoom. In the case of the P_606_’::mKO-tetON, *smyc’*::mCherry reporter strain infection, as indicated in the Results section, the lack of apparent doxycycline penetration into the central core precluded analysis of the reporter signal of the bacteria residing there, and thus in this case, high magnification and resolution images for reporter signal quantification of Mtb in the lesion core were taken only from the more peripheral regions of the core. Images for reporter quantification of Mtb residing in the lesion cuff were taken from areas containing bacteria in the CD68-positive region ringing the lesion core. As with the images from the core, regions were selected based only on bacteria presence, and without regard for reporter signal. To further ensure robustness of data, at least 5 lesions across multiple mice were analyzed.

Quantification of reporter signal was carried out essentially as previously described using Volocity software [13,25,67]. In brief, for the *rv2390c’*::GFP reporter, the volume of each bacterium was measured via the mCherry channel, and the corresponding total GFP signal for that given object (bacterium) simultaneously measured. The settings for the GFP channel were maintained across samples to allow for comparison of values. Statistical analysis was performed using a Mann-Whitney test for comparison of reporter signal in bacteria present in the cuff versus core region in each lesion. A multiple t-test with a Holm-Sidak correction was used for statistical analysis of the binned data of reporter signal in the cuff versus core across all lesions. The P_606_’::mKO-tetON, *smyc’*::mCherry reporter strain was quantified in the same manner as for the *rv2390c’*::GFP reporter. For the SSB-GFP reporter, individual bacteria were identified via the mCherry channel and the number of bacteria with SSB-GFP puncta determined. Numbers of bacteria quantified in each case are indicated in the figure legends. Statistical analysis was performed using a Wilcoxon matched-pairs signed rank test for comparison of lesion cuff versus core values, and with a Mann-Whitney statistical test for comparing drug to mock treatment in a lesion sublocation.

## Supporting information

S1 Fig Bacterial load in C3HeB/FeJ murine infection model. C3HeB/FeJ mice were infected with Mtb for 6 or 8 weeks, before sacrifice and lung homogenates plated for colony forming unit determination. Each point represents a single mouse. Horizontal lines indicate the median.
(TIF)
