# Supplementary figures and images for: Spatial relationships of intra-lesion heterogeneity in Mycobacterium tuberculosis microenvironment, replication status, and drug efficacy
Source: PLoS Pathog. 2022 Mar 28;18(3):e1010459. doi: 10.1371/journal.ppat.1010459 (PMC8989358; doi:10.1371/journal.ppat.1010459)

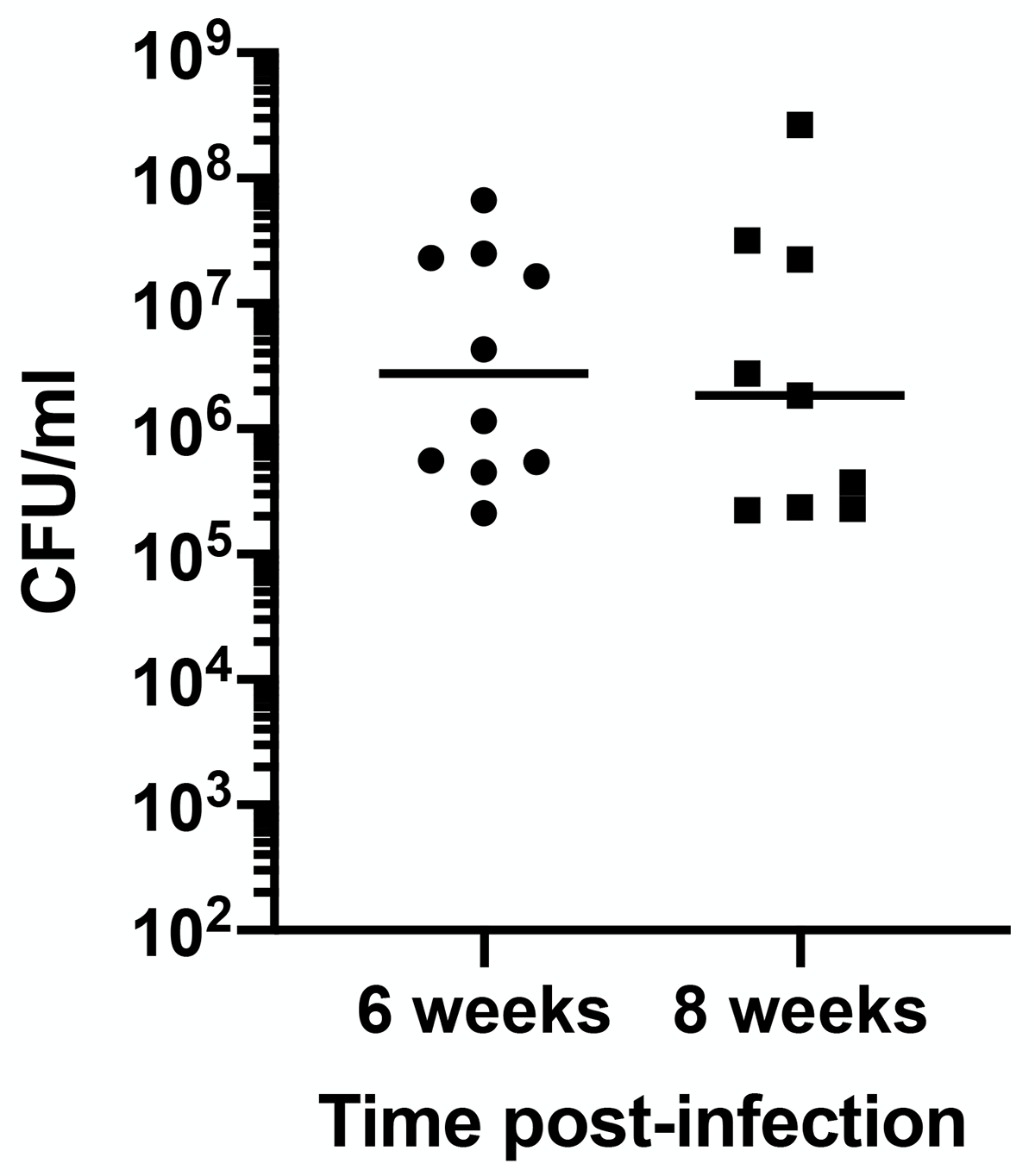

Supplement: S1 Fig — C3HeB/FeJ mice were infected with Mtb for 6 or 8 weeks, before sacrifice and lung homogenates plated for colony forming unit determination. Each point represents a single mouse. Horizontal lines indicate the median. (TIF) [file ppat.1010459.s001.tif]
